# Duodenal carcinoma at the ligament of Treitz. A molecular and clinical perspective

**DOI:** 10.1186/1471-230X-10-109

**Published:** 2010-09-17

**Authors:** Peter T Kalogerinis, John E Poulos, Andrew Morfesis, Anthony Daniels, Stavroula Georgakila, Thomas Daignualt, Alexandros G Georgakilas

**Affiliations:** 1 Methodist University Physician Assistant Program, Fayetteville, North Carolina, USA; 2 Fayetteville Gastroenterology Associates, Fayetteville, North Carolina, USA; 3 Owen Drive Surgical Clinic of Fayetteville, Fayetteville, North Carolina, USA; 4 Harris Birthright Research Centre for Fetal Medicine, Department of Obstetrics and Gynecology, Kings College University Hospital, London SE5 9SR, UK; 5 Department of Biology, Thomas Harriot College of Arts and Sciences, East Carolina University, Greenville, North Carolina 27858, USA

## Abstract

**Background:**

There is very small occurrence of adenocarcinoma in the small bowel. We present a case of primary duodenal adenocarcinoma and discuss the findings of the case diagnostic modalities, current knowledge on the molecular biology behind small bowel neoplasms and treatment options.

**Case:**

The patient had a history of iron deficiency anemia and occult bleeding with extensive workup consisting of upper endoscopy, colonoscopy, capsule endoscopy, upper gastrointestinal series with small bowel follow through and push enteroscopy. Due to persistent abdominal pain and iron deficiency anemia the patient underwent push enteroscopy which revealed adenocarcinoma of the duodenum. The patient underwent en-bloc duodenectomy which revealed T3N1M0 adenocarcinoma of the 4th portion of the duodenum.

**Conclusions:**

Primary duodenal carcinoma, although rare should be considered in the differential diagnosis of occult gastrointestinal bleeding when evaluation of the lower and upper GI tract is unremarkable. We discuss the current evaluation and management of this small bowel neoplasm.

## Background

Malignancies of the small intestine are uncommon, accounting for only roughly 1-2 % of malignant gastrointestinal (GI) diseases [1]. When compared to other cancer diagnosis rates, small bowel cancers average roughly 6000 per year in the United States [2]. As suggested by two recent major epidemiological studies on patients with small bowel neoplasms (SBN) identified from the National Cancer Data Base (NCDB, 1985-2005) and the Surveillance Epidemiology End Results (SEER, 1973-2004) database [3] as well as the Connecticut Tumor Registry [4], over the past twenty years, carcinoid tumors have become the most common SBN followed by adenocarcinomas (AC). A significant observation based on these studies is that from 1973 to 2004, the incidence of carcinoid tumors increased more than 4-fold (2.1 to 9.3 per million), with similar increases in the incidence of AC, stromal tumors, and lymphomas [3]. While AC is the most common malignancy of the duodenum the most common site of SBN is the ileum (Table 1), with a preponderance of lymphoma and carcinoids [5]. Among patients with Crohn's disease AC is most noted in ileum rather than the more proximal small bowel [6]. AC of the 3^rd ^and 4^th ^portions of the duodenum is very uncommon [7], and only 45% of duodenal carcinomas occur in that region [8].

**Table 1:** Accumulating reports on the incidence of small bowell malignancies: type, location and survival rates

Incidence (%) of small bowel malignancy based on histology				
**Adenocarcinoma**	**Carcinoid** **Tumors**	**Lymphoma**	**Sarcoma**	**Reference**
40	25	25	10	[69]
47	28	12	13	[70]
40	20	27	9	[71]
36.9	37.4	17.3	8.4	[4]
27	33	16.3	7.1	[3]

**Incidence (%) of small bowel adenocarcinoma based on location**				

	**Duodenum**	**Jejunum**	**Ileum**	**Reference**
	72	37	21.4	[69]
	47	29	24	[70]
	41	34	25	[71]
	56	15.6	13	[4]
	53	19.7	12.9	[3]
				

**Incidence (%) of small bowel tumors by location**				

	**Duodenum**	**Jejunum**	**Ileum**	**Reference**
	24.6	36.9	38.3	[69]
	32	36	32	[70]
	23	33.3	41.6	[71]
	33	12	26	[4]
	25	15.3	29.7	[3]
				

**Five year survival rate (%) of small intestine adenocarcinoma based on disease stage **[2]				

Stage I	55			
Stage II A	49			
Stage II B	35			
Stage III A	31			
Stage III B	18			
Stage IV	5			

The low incidence of SBN may be due to several theoretical factors including small bowel transit time, host immunologic factors, and/or epithelial toxin exposure [9-11]. Dietary factors that may increase the risk of small bowel AC may include diets high in red meat, or the consumption of smoked or salted foods [12]. There may be an increased risk of SBN with a diet rich in refined carbohydrates, and sugar [13]. Hereditary syndromes or conditions that can predispose to SBN include Muir-Torre syndrome [14], hereditary nonpolyposis colorectal cancer (HNPCC), familial adenomatous polyposis (FAP) and it's variants such as Gardner's Syndrome [15] Celiac Sprue, Puetz-Jeghers, Crohn's Disease[16] and Juvenile Polyposis Syndrome [17].

Primary SBNs are much rarer than those that arise from a secondary neoplastic process [16]. Metastasis from the stomach, ovary, colon and uterus can involve the small bowel by direct means or via peritoneal involvement [18]. Metastatic tumors from breast, melanoma and lung appear to spread to the duodenum by blood and lymphatic pathways.

The mean age of presentation of SBN is 64 with a range of 47-87 years [3,19]. Obscure GI bleeding (OGIB) is the most common symptom as 50% of those with SBN present with OGIB, however it should be noted that only 4% of OGIB cases are caused by SBN [20]. Due to the vague presentation a delayed diagnosis or misdiagnosis is common [9], with an average delay of six to eight months between the time of symptom onset and diagnosis [21].

### Investigations

SBN are usually discovered during the evaluation of OGIB, anemia, and abdominal pain. Abdominal X-ray may help in showing obstruction, however duodenal carcinomas especially those in the 3^rd ^and 4^th ^portions of the organ are often missed on barium x-ray examination [22] yielding a definite diagnosis in less than 5% of cases [23]. Colonoscopy with ileoscopy may be useful in detecting lesions in the terminal ileum and excluding a colonic source of pathology. Both sporadic duodenal adenomas and those associated with hereditary cancer syndromes have a higher risk of colorectal cancer (CRC) and these patients should be evaluated with colonoscopy [24]. Likewise those with CRC associated with hereditary cancer syndromes should be evaluated for SBN [17].

The utilization of CT enterocolysis (CTE) in the detection of SBN overcomes the individual short comings of both barium enterocolysis and conventional CT and utilizes the advantages of both into a single technique and has begun to substitute enterocolysis in clinical practice [25]. Contrast-enhanced and water-enhanced multidetector CTE has a sensitivity of 84.7-95% and 96-100 % specificity for the detection of SBN [26,27].

Tocchi *et al*. found that upper GI endoscopy had a 36% false-negative result rate in identifying duodenal tumors due to depth of insertion. Push enteroscopy (PE) provides many benefits including direct visualization of lesions in the proximal duodenum and jejunum, allowing the ability to biopsy and provide therapeutic measures in cases of bleeding. The investigation of obscure bleeding by PE may find a diagnostic cause in 25-28% of cases [28,29]. PE and Sonde enteroscopy have shown a diagnostic yield of 6% for SBN in patients undergoing the procedure for evaluation of OGIB [30]. However PE as well as CT and small bowel barium studies may fail to detect 50% of small bowel lesions [31].

Capsule endoscopy (CE) has been shown to be a safe and effective non invasive method of diagnosis for small bowel abnormalities [32,33] and allows a more detailed inspection of the small intestine. CE has also been shown to detect duodenal adenomatous polyps in 64.3% of those who also have FAP [34]. An absolute contraindication to CE is GI obstruction. Relative contraindications to CE include pregnancy, GI motility disorders, or large diverticuli within the small bowel [35]. CE may detect more SBN than CTE in patients with OGIB having an overall accuracy of 84.7%[36].

It has been shown that CE diagnosed SBN in 9% of patients who underwent the procedure for investigation of OGIB and in 8.3% of those who were investigated for non bleeding causes [37]. However in a pooled meta-analysis it was found that CE had a 20% miss rate for SBN [38]. Similar to our case where CE failed to reveal AC of the duodenum, there are increasing reports in the literature of failure of CE to detect solitary SBN [39,40]. It has also been shown that after an initial negative CE study a repeat CE may reveal significant lesions in 20% of cases [41]. Etiologies for failure to detect lesions by CE may be due to rapid capsule passage through the proximal small bowel, decreased visibility due to luminal contents, or failure to reach the colon. Thus, based on certain clinical scenarios a negative finding on CE may not exclude significant small bowel pathology and further investigation may be warranted.

Balloon assisted enteroscopy (BAE) utilizing either single balloon enteroscopy (SBE) or double balloon enteroscopy (DBE) offers a number of advantages when compared to other small bowel imaging studies. The advantages include visualization of the entire small bowel with the ability to provide tissue diagnosis and provide therapeutic modalities such as control of bleeding and dilation of strictures [42,43]. Optimal visualization of the small bowel may involve both oral and anal insertion. Initial studies indicated a greater diagnostic yield and higher rate of endoscopic intervention for DBE vs. SBE[44]. However a recent study comparing SBE vs DBE revealed identical procedure times, depth of insertion, and a slight increase in identification and treatment of lesions with SBE vs DBE[45]. Studies have calculated that BAE and CE are in agreement 61-74% of the time and 96% of the time when diagnosing large tumors [46]. In regards to SBN, BAE can often find lesions originally missed by CE and is suggested as a follow up study to a negative CE exam [47]. Arakawa reported equal diagnostic yields for both CE and BAE with false negative cases of CE and BAE due to failure to detect lesions in the proximal small bowel and inaccessibility of the site, respectively. In a recent meta-analysis comparing CE and BAE, there was no significant difference in yields between the two modalities 61% vs. 56%, respectively[48]. Sub analysis of data did reveal a slight advantage in favor of CE and this appeared to be to the utilization of a single insertion approach by BAE. When BAE was performed using a dual insertion approach via the oral and anal route the yield was 74% vs. 54% for CE [48].

The failure of BAE to show superiority over CE in the detection of lesions may be due to complete evaluation of the entire small bowel in only 60-70% of cases [43,49]. A disadvantage of the procedure is the time needed to visualize the small bowel [50], its invasiveness, and the reports of intestinal necrosis [51], perforation and acute pancreatitis [52] post procedure. Due to the failure of a true gold standard in evaluation of the small bowel utilization of both these procedures may be complementary.

### Treatment and Prognosis

Duodenal AC has a shorter median overall survival rate compared with patients with tumors located in the jejunum or ileum [53]. SBNs are more common in men [54] and are higher in African Americans than those of Caucasian decent. It has been reported that SBN in African American men has increased in prevalence by 120% over the last 3 decades [55]. In regards to 5 year survival, earlier stages have a better prognosis [56]. Around 58% of patients with small intestine AC present at late stages (III and IV), in contrast with 28% of patients with CRC[55] (Table 1). The overall median survival of patients with duodenal AC has been reported as 18 months and the 5-year survival as 23% [57].

Historically treatment of SBN has relied solely upon surgery as the only curative treatment and has been divided between two techniques which are pancreatoduodenectomy (PD) and duodenal segmentectomy (DS). PD is considered to be the procedure of choice. DS, is used for more palliative measures [8]. Studies have shown that DS is a better option for distal duodenal tumors without advanced disease, in which case PD is considered a better option [8,57,58]. Surgical intervention has shown to provide a curative resection in 40-65% of patients. The five year survival rate for non-resected tumors being is 15-30% compared to 40-60% survival rate for those who had resection [53]. A large tumor or positive lymph node metastasis does not invalidate resection as long as a negative margin can be attained, and in terms of clearance of regional lymph nodes the difference between both procedures is negligible [57].

Chemotherapy is mainly utilized as a palliative measure and has not been well studied due to the low prevalence of AC in the small bowel. The largest published study investigating chemotherapeutic measures for small bowel AC involved 14 subjects with metastatic small bowel AC and involved a chemotherapeutic regiment containing 5-fluorouracil (5-FU) [59]. Patients had a median survival of 9 months. A more recent investigation reported advanced small bowel AC treated with infusional 5-FU-based regimens had a response rate of 37.5% and a median survival of 13 months [60]. A case report using onastat, tegafur, and gimestat (otherwise known as S-1chemotherapy) showed remission of primary AC of the duodenum [61]. Newer agents found to be effective for CRC also may have an effect on small bowel AC.

### Genetic and Molecular Biology Considerations

Due to the rarity of SBN, little has been published about oncogenesis as well as clinicopathologic features [62]. An analysis of SBN found that 53% had point mutations in the Ki-*ras *gene [63] similar to mutations found in CRC[64], and that overall frequencies of Ki-*ras *and *p53 *gene mutations are similar in both [63].

In terms of the APC gene, SBN have a lower rate of mutations involving the APC gene compared to its involvement in CRC [63]. Duodenal carcinoma is the second most common carcinoma in FAP and the low rate of APC mutations in duodenal adenocarcinoma refer primarily to sporadic adenocarcinomas and not those associated with FAP and its variants. Thus these recent findings suggest that the APC gene is not involved with SBN in man [63,65]. An extensive study revealed all duodenal AC tumors to be positive for mismatch repair (MMR) on genes hMHL1 and hMSH2 but no mutations were found in the mutation cluster region (MCR) of the APC gene [66]. Thus suggesting that molecular mechanism leading to the development of AC of the small intestine may be different than those leading to CRC. Cytogenetical studies on primary duodenal AC revealed several abnormalities that resulted in partial or complete losses or gains chromosomally [67]. The detection of biallelic MMR gene mutations in pediatric duodenal cancer further supports the idea of MMR deficiencies as a duodenal cancer predisposition syndrome [68].

## Case Presentation

A 66 year old African-American female presented with complaints of 10 pound weight loss and a four week history of intermittent abdominal pain, nausea, and non bloody emesis. Her medical history was significant for type 2 diabetes mellitus, hypertension, coronary heart disease, and peptic ulcer disease. The patient denied any significant alcohol or tobacco use. Her family history was positive for colon cancer. Physical exam and laboratory tests were unremarkable. A colonoscopy and an esophagogastroduodenosopy (EGD) to the second portion of the duodenum were performed revealing three small tubular adenomas of the colon and helicobacter pylori gastritis. She was treated with two weeks of amoxicillin 1 gram and clarithromycin 500 mg orally twice daily for two weeks.

She did well after the antibiotic therapy and was not seen again until two years later when she was hospitalized for severe, symptomatic anemia. For eight months prior to admission, she noted recurrent intermittent abdominal pain and nausea with non-bloody emesis and progressive fatigue. She denied melena or hematochezia. On admission, her hemoglobin was 5.4 gm/dl and hematocrit was 17.3%, with a normal MCV. Her stool was hemoccult positive. She required a transfusion of four units of packed red blood cells. An EGD to the second portion of the duodenum revealed mild gastritis, negative for *H. pylori*. A CT scan of the abdomen and pelvis with oral contrast was unremarkable. An outpatient wireless capsule endoscopy was ordered; however, it was cancelled due to the reluctances of the patient to swallow the capsule.

Patient again required hospitalization for severe symptomatic anemia with hemoglobin of 5 gm/dl. Her indices and iron studies at this time were consistent with iron deficiency anemia. She denied melena, hematochezia or bloody emesis. She required another 4 unit blood transfusion. An EGD was performed and again it was unremarkable. Due to her inability to swallow the wireless capsule, the endoscope was used to deliver the capsule into the stomach. The study however was limited due to retained debris in the mid duodenum, significantly limiting visualization of the small bowel.

Patient presented three months later with symptomatic anemia, hemoglobin of 4.6 gm/dl and hematocrit of 14.2%. She required four units of packed red blood cells. Two days prior to admission, she noted black tarry stools. Physical exam was unremarkable with the exception of palpable tenderness in the epigastric and left upper quadrant.

Three way abdominal x-ray of the abdomen was performed and was unremarkable. MRA with and without contrast and CT scan of abdomen and pelvis with oral contrast showed no evidence of localized abnormality in the abdomen or pelvis in terms of solid organs or vasculature. An upper GI series with KUB was performed revealing eccentric broadband defect along the inner or medial wall of the 2nd portion of the duodenum, with the other portions being unremarkable.

**Figure 1:**
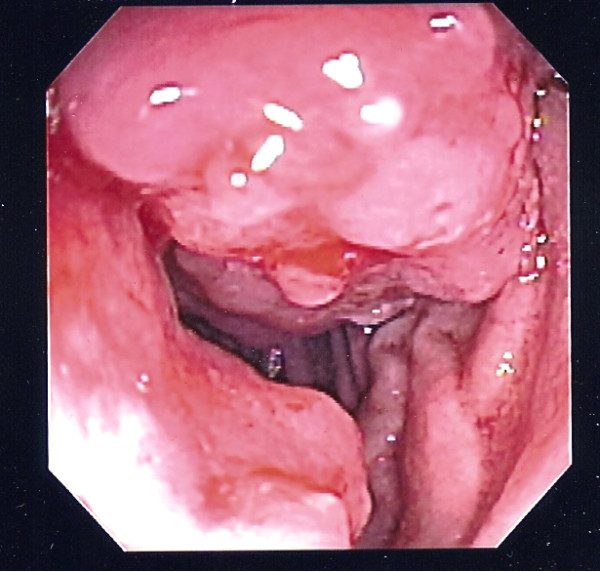
**Endoscopic image of the small bowel neoplasm**. The endoscopic image of the cancerous mass showing it's large irregular pattern and causing narrowing of the duodenum.

Push enteroscopy was performed which revealed a circumferential fungating mass in the 4th portion of the duodenum, which was actively oozing blood and upon further investigation it appeared the mass extended to the ligament of Treitz (Figure 1). The area was biopsied and tattooed. Pathology from biopsy revealed moderately differentiated AC with lymphovascular invasion. Patient underwent exploratory laparotomy and the small bowel was examined with the tumor being present at the ligament of Treitz. The tumor was resected en bloc and two lymph nodes were collected. The small bowel was reconnected using a primary Gambee anastamosis.

Pathology from surgical specimen revealed T3N1M0 AC, with the tumor being 4.5 centimeters in greatest dimension and showing invasion through the muscularis propria and into the sub-serosa but not through it. The resected margins were clear. Of the two lymph nodes collected one was positive for metastatic carcinoma, with the tumor nodule measuring 1.5 centimeters in diameter and showing invasion through the lymphatic capsule.

Patient was referred to oncology for consultation however did not follow up as scheduled, and has been lost to follow up care.

## Conclusions

In patients presenting with OGIB, iron deficiency anemia or other warning signs and symptoms SBN, should be considered in the differential due to its insidious presentation. In terms of oncogenesis more research is needed in order to better understand its development, but evidence suggests a multi-factorial genetic cause. Options in the evaluation of small bowel pathology may require CE, BAE, and/or CTE. An initial approach may be with CE or CTE due to the fact it is non invasive with subsequent utilization of BAE if the evaluation is unrevealing or if lesions are detected that require tissue confirmation. Surgery is the best curative option in terms of treatment of these types of malignancies with PD being better for advanced diseases and DS for disease of the distal duodenum. Chemotherapeutic options are improving and providing longer survival rates and palliative benefits.

## Consent

Written informed consent was obtained from the patient for publication of this case report and any accompanying images. A copy of the written consent is available for review by the Editor-in-Chief of this journal.

## Abbreviations

GI: Gastrointestinal; SBN: Small bowel neoplasms; NCDB: National Cancer Data Base; SEER: Surveillance Epidemiology End Results; AC: Adenocarcinoma; HNPCC: hereditary nonpolyposis colorectal cancer; FAP: familial adenomatous polyposis; OGIB: Obscure GI bleeding; CRC: Colorectal cancer; CTE: CT enterocolysis; PE: Push enteroscopy; CE: Capsule endoscopy; BAE: Balloon assisted enteroscopy; SBE: Single Balloon enteroscopy; DBE: Double Balloon enteroscopy; PD: pancreatoduodenectomy; DS: duodenal segmentectomy; 5-FU: 5-fluorouracil; MMR: Mismatch repair; MCR: mutation cluster region; EGD: esophagogastroduodenosopy

## Competing interests

The authors declare that they have no competing interests.

## Authors' contributions

FAM, JEP, TD, and AD contributed directly and equally to patient care; PTK, SG, AGG, and JEP contributed to literature research and analysis of the data; All authors contributed in the writing and critical development of the manuscript. All authors have read and approved the final manuscript.

## Pre-publication history

The pre-publication history for this paper can be accessed here:

http://www.biomedcentral.com/1471-230X/10/109/prepub
